# An Unusual Location for Orbital Schwannoma: A Case Report

**DOI:** 10.1002/ccr3.70567

**Published:** 2025-06-02

**Authors:** Mohammad Etezad Razavi, Mehrdad Motamed Shariati, Mostafa Fakoor

**Affiliations:** ^1^ Eye Research Center Mashhad University of Medical Sciences Mashhad Iran

**Keywords:** extraocular muscle, orbital schwannoma, peripheral nerve sheath tumors, proptosis

## Abstract

Orbital schwannomas are benign and slow‐growing tumors that constitute 1% of all orbital tumors. They arise from the sensory branch of the ophthalmic division of the trigeminal nerve and are hence more often found in superior orbit regions. In this case report, we presented a 34‐year‐old healthy man with proptosis in the right eye and diplopia. Imaging revealed a large, well‐circumscribed mass in the right medial rectus. The patient underwent surgical resection of the tumor, and the patient's symptoms resolved 5 days after surgery. Pathological and immunohistochemical evaluations confirm the diagnosis of schwannoma.

## Graphical abstract

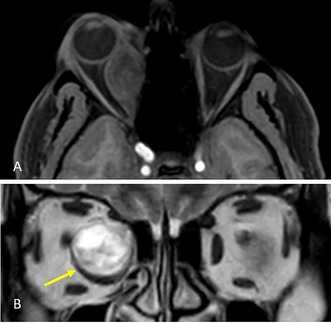

**Summary**

- Orbital schwannomas rarely involve extraocular muscles.
- This case highlights the importance of including schwannoma in the differential diagnosis of rectus muscle tumors.
- Prompt surgical resection can result in excellent functional recovery with minimal complications, even when the tumor arises from within an extraocular muscle.

## 1 Introduction

Schwannoma, also known as neurilemmoma, is a rare tumor originating from Schwann cells in the peripheral nerve sheath [1]. This tumor was described by Verocay in 1910 and Antony in 1920. Schwannoma may be found all over the body but is most commonly found in the head and neck. This tumor accounts for 1% of all orbital tumors [2]. Most orbital schwannomas originate from the sensory branch of cranial nerve V. Supraorbital and supratrochlear nerves are commonly affected and are more often found in superior orbital regions. Schwannoma less commonly originates from motor nerves such as oculomotor or ciliary nerves [3, 4]. This tumor usually affects adults between 20 and 50 years old and commonly presents as painless, insidious ocular proptosis, globe displacement, diplopia, or compressive optic neuropathy [1, 2, 3, 4].

This case report presents a patient with gradually progressive proptosis and limitation of adduction in the right eye, who was finally diagnosed with an orbital Schwannoma located in the medial rectus muscle.

## 2 Case History/Examination

A 34‐year‐old healthy man presented with gradually progressive proptosis since a year ago, with diplopia and decreased vision in the right eye. The best‐corrected distance visual acuity (BCDVA) was 20/25 and 20/20 in the right and left eyes, respectively. The pupillary reaction was normal, and there was no relative afferent pupillary defect (RAPD). The intraocular pressure (IOP) was within normal limits for both eyes. In the slit lamp examination, we found no remarkable findings. Fundus examination showed no pathology. In the external examination, axial proptosis and limitation of adduction in the right eye were apparent.

## 3 Methods

### 3.1 Radiological Evaluation

**FIGURE 1:**
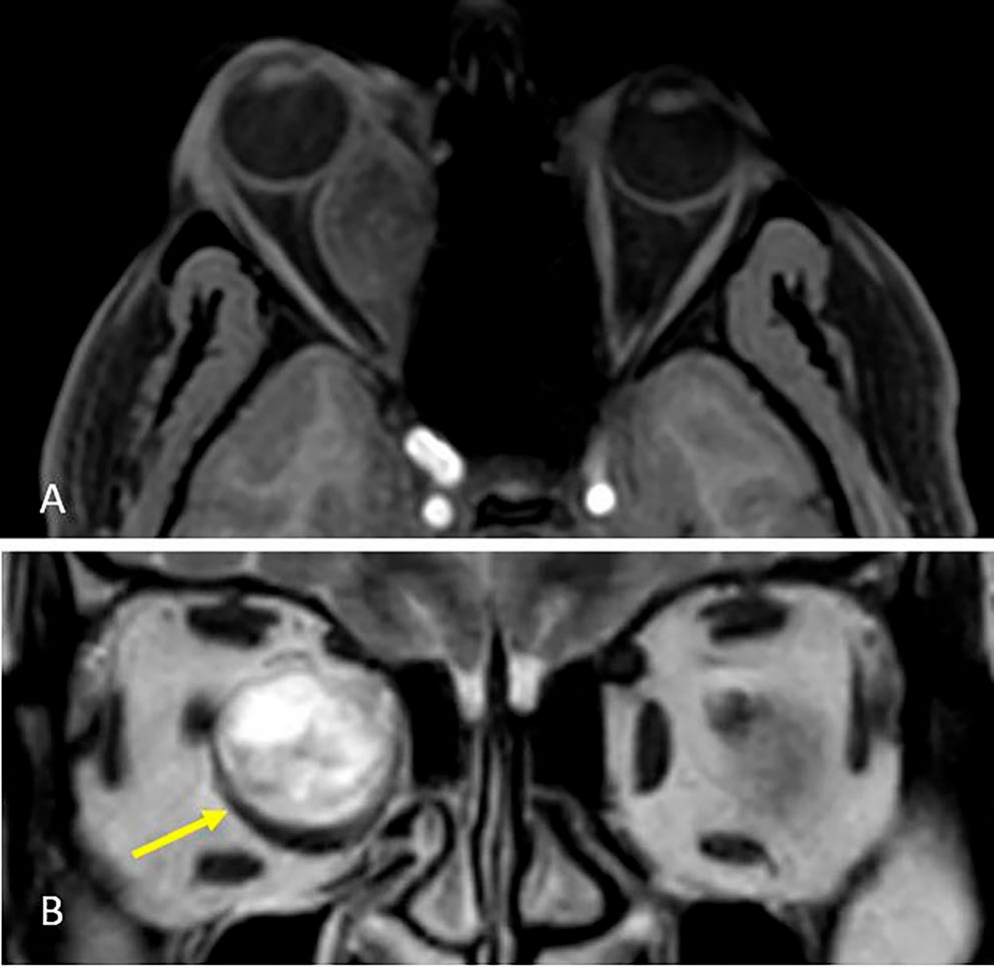
MRI. (A) T1‐weighted with contrast showed a large orbital mass heterogeneously enhancement and cystic space. (B) T2WI showed large hyperintense mass displace medial rectus (yellow arrow).

After the examinations, an orbital MRI was performed for the patient, and magnetic resonance imaging revealed a large well‐circumscribed mass located in the right medial rectus that was uniformly enhanced with a cystic component and a heterogeneously hyperintense lesion in T2WI (Figure 1).

### 3.2 Surgical Intervention

**FIGURE 2:**
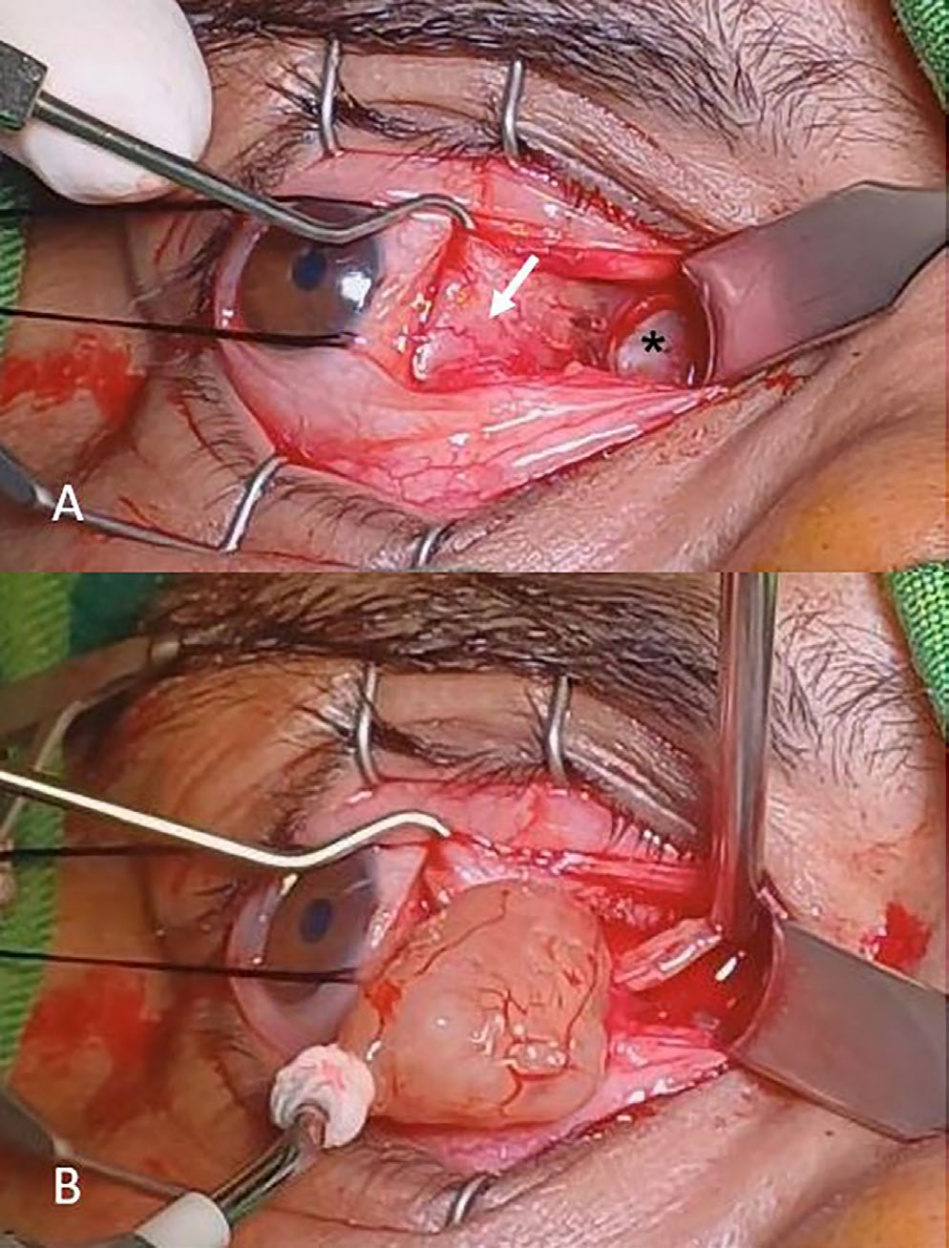
Intraoperative photograph. (A) Mass (asterisk) attached to medial rectus (white arrow). (B) Brown‐cream mass removed with cryoprobe.

The tumor was resected via the transcaruncular approach. After hooking the medial rectus muscle and visualization of the tumor, the tumor was carefully separated from the muscle using blunt dissection. The tumor was located in the medial rectus muscle, and after blunt dissection and separation from the muscle, it was removed with a cryoprobe (Figure 2). After removing the tumor, a search was made to remove its remains, and hemostasis was performed with cauterization. This stage was conducted with meticulous care to prevent damage to surrounding tissues as well as the muscle itself. Subsequently, a thorough examination of the medial rectus muscle was performed, revealing no signs of serious injury that would necessitate immediate intervention. At the end of the procedure, an absorbable suture was used to close the conjunctiva.

### 3.3 Postoperative Examination

**FIGURE 3:**
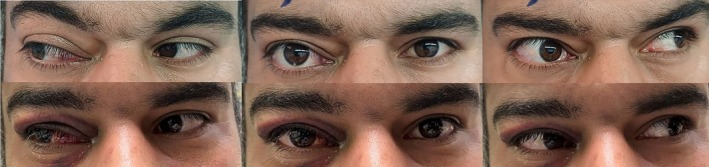
External photograph of patient. Top row: proptosis and limitation of adduction in right eye. Bottom row: 5 days after surgery resolved proptosis and limitation (published with patient's written informed consent).

After surgery, the patient was treated with betamethasone drops every 4 h and chloramphenicol drops every 6 h for 2 weeks. At the 5‐day post‐operation follow‐up, proptosis and limitation of adduction in the right eye resolved, and eye movement in all directions was normal. Visual acuity in both eyes improved to 20/20 (Figure 3).

### 3.4 Histopathological Examination

**FIGURE 4:**
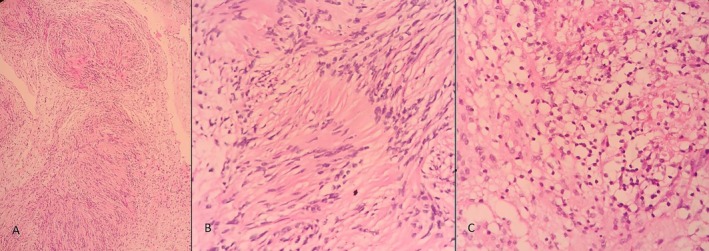
Histopathological evaluation of tumor. (A) H&E stain showed Antoni A and Antoni B regions (×10). (B) Antoni A regions with nuclear palisading (×40). (C) Antoni B regions (×40). H&E, hematoxylin and eosin.

**FIGURE 5:**
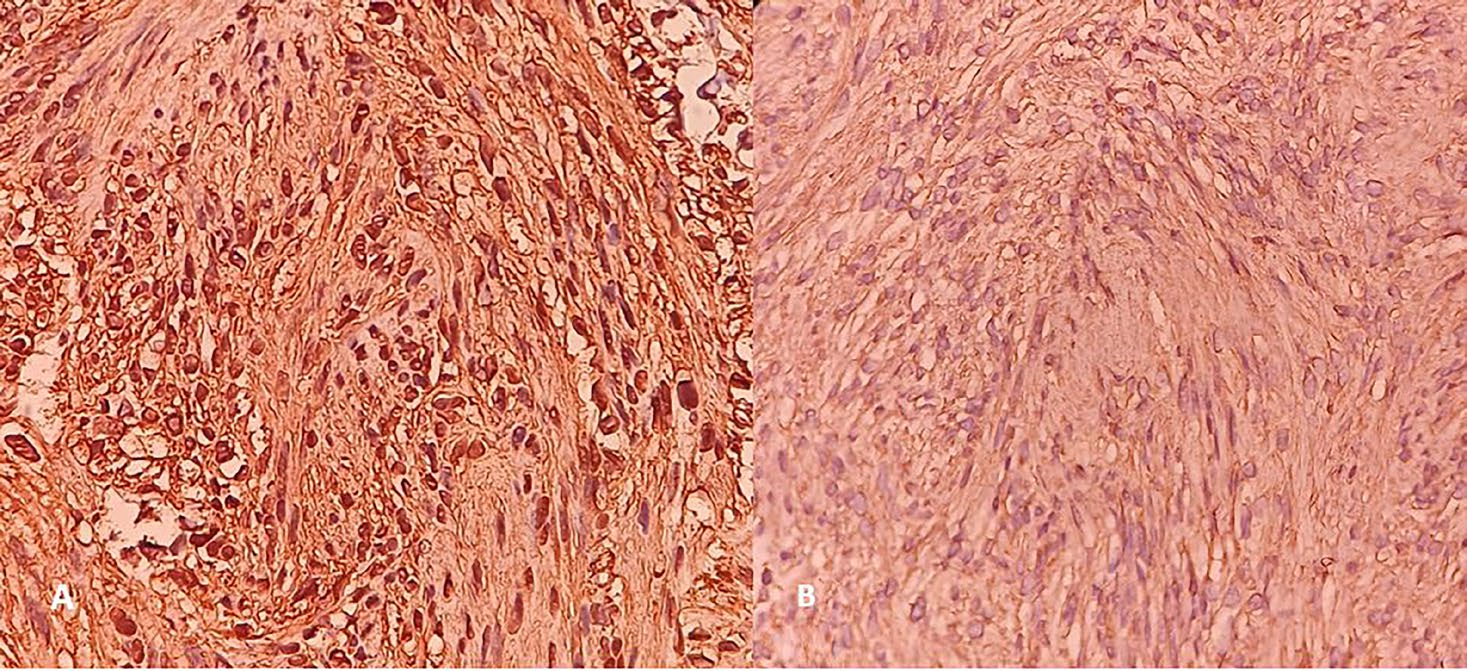
Immunohistochemical evaluation of tumor. (A) S100 positive in spindle cells (×40). (B) Vimentin positive in spindle cells (×40).

A well‐circumscribed brown‐cream tissue approximately 17 mm in diameter was excised from the medial rectus muscle. Histopathological evaluation showed a spindle‐cell tumor with palisading patterns and a cystic degeneration zone (Figure 4). Immunohistochemical evaluation was positive for vimentin and S100 (Figure 5).

## 4 Discussion

Schwannomas are one of the peripheral nerve sheath tumors (PNST) found throughout the body. They are benign, slow‐growing nerve sheath tumors that originate from Schwann cells and occur in women slightly more than men [1, 2, 3, 4, 5]. Schwannomas are rare tumors that occur in adults between 20 and 50 years old and account for 1% of orbital tumors [1]. Orbital schwannoma usually originates from the first branch of the trigeminal nerve and is located in the superior region of the orbit; however, it can arise from the peripheral branch of the oculomotor, trochlear, abducens nerves, parasympathetic, and sympathetic nerves, and ciliary ganglion [3, 4, 6, 7]. In a review of 15 patients with orbital schwannomas by Pointdujour‐Lim et al., in 11 cases, the tumor was in the superior orbit, and in four cases, it was in the inferior orbit. The tumor was intraconal, extraconal, or both, and anteroposterior extension was the anterior third, middle third, posterior third, or entire of the orbit [8].

Due to the complexity of the orbital structure, it is difficult to determine the specific origin of the tumor [6]. They are asymptomatic when small, but slowly grow and cause symptoms such as proptosis, diplopia, and reduced vision, which need treatment in these cases [2, 9, 10].

In growth pathology, they are solitary light‐tan color tumors surrounded by a thin fibrotic capsule. Yellow color is common due to lipid content and lipid‐laden macrophages. Cyst and hemorrhagic changes may be present. On light microscopy, classic schwannoma has two patterns of cell morphology: compact patterns (Antoni A) and loose patterns (Antoni B). Antoni's type‐A zone is characterized by packed spindle cells with fusiform nuclei and eosinophilic cytoplasm. Where in this zone, palisading patterns called Verocay bodies may be encountered. Antoni's type‐B zone consists of loosely packed myxoid cells with a disorganized arrangement [11]. Immunohistochemical staining of schwannomas is positive for S‐100, Vimentin [1].

Diagnosing orbital schwannomas is challenging through imaging alone and requires histopathological evaluation. On computed tomography (CT) orbital schwannoma appears heterogeneously iso to hypodense and shows moderate enhancement in post‐contrast images. On magnetic resonance imaging (MRI), imaging findings correlate with two patterns observed in histopathology. Tumors with dominant Antoni A zones are solid, and tumors with dominant Antoni B zones are cystic and more vascular. On T2WI, the Antoni A zone is a slightly high signal and is significantly high in the Antoni B zone. On post‐contrast images, the Antoni B pattern has significantly stronger enhancement than the Antoni A pattern. T1 weighted images have a nonspecific pattern of heterogeneous iso to hypo intensity [1, 8, 12].

Various differential diagnoses have been reported for orbital schwannoma in the literature. We review the clinical presentation, imaging findings, and histopathologic characteristics of various orbital tumors in Table 1 [13, 14].

**TABLE 1:** Differential diagnosis of orbital tumors.

	Clinical features	Radiologic features	Histopathology and immunohistochemistry
Orbital schwannoma	Painless proptosis, diplopia, visual changes	Well‐circumscribed, iso‐ or hypointense on T1, hyperintense on T2	Antoni A and B patterns encapsulated S‐100 positive
Cavernous hemangioma	Painless, slowly progressive proptosis	Well‐circumscribed, homogeneous, enhancing	Vascular channels, no Antoni patterns, encapsulated S‐100 negative
Meningioma	Slowly progressive vision loss, proptosis	Uniform enhancement, bony hyperostosis	Whorled pattern, psammoma bodies EMA positive, S‐100 negative
Neurofibroma	Proptosis, associated with NF1 in some cases	Diffuse, infiltrative mass	A mixture of Schwann cells, fibroblasts, no Antoni patterns S‐100 positive, heterogenous
Solitary fibrous tumor	Painless, slowly progressive proptosis	Well‐circumscribed, hypervascular	Spindle cells in a collagenous stroma CD34 positive, S‐100 negative
Inflammatory pseudotumor	Pain, proptosis, diplopia, signs of inflammation	Diffuse, infiltrative mass	Mixed inflammatory infiltrate

Although orbital schwannomas most commonly arise from the sensory branches of the ophthalmic division of the trigeminal nerve, involvement of extraocular muscles is exceedingly rare. Only a few cases of intramuscular orbital schwannomas have been reported in the literature. Van Horn et al. described a case of intramuscular schwannoma with cystic degeneration located within the medial rectus muscle in a patient with neurofibromatosis type 2, highlighting its progressive changes in MRI characteristics over several years [15]. Similarly, Afshar et al. reported a schwannoma arising from the inferior rectus muscle in a young woman, emphasizing the importance of including schwannoma in the differential diagnosis of orbital tumors located within extraocular muscles [16]. In our case, the tumor was located in the medial rectus muscle and, based on its location, it was most likely derived from a branch of the oculomotor nerve that innervates this muscle.

The main treatment of orbital schwannoma is surgical excision. The surgical approach varies depending on the anatomical location and extent of the tumor. To prevent tumor recurrence, complete surgical resection has to be considered. Schwannomas can be resected totally from the originating nerve because of its peripheral outpouching growth pattern [1, 2, 3, 6, 9, 17].

## 5 Conclusion

In our case, schwannoma was confirmed in pathology and immunohistochemistry. This case is specific because of the location of the tumor. The tumor was located in the right medial rectus and was most likely derived from a branch of the oculomotor nerve that supplies this muscle. Although the tumor was large and displaced the medial rectus, 5 days after surgery, proptosis and limitation in ocular movement resolved. The patient was followed up 3 months after the surgery and did not mention any particular problems.

This study is limited by its single‐case design, which may not be representative of all orbital schwannomas involving extraocular muscles. The lack of long‐term follow‐up data restricts conclusions regarding recurrence or late complications.

In this case, it is emphasized that schwannoma can involve different areas of the orbit and it should be considered in the differential diagnosis of orbital tumors.

## Author Contributions

**Mohammad Etezad Razavi:** conceptualization, supervision. **Mehrdad Motamed Shariati:** writing – review and editing. **Mostafa Fakoor:** data curation, investigation, writing – original draft.

## Consent

Written informed consent was obtained from the patient to publish this case report and any accompanying images. A copy of the written consent is available for review by the Editor‐in‐Chief of this journal.

## Conflicts of Interest

The authors declare no conflicts of interest.

## Data Availability

The datasets used during the current study are available from the corresponding author upon reasonable request.
